# In Honor of Professor Zhifang Chai

**DOI:** 10.3390/molecules29194627

**Published:** 2024-09-29

**Authors:** Dongqi Wang, Zhiyong Zhang

**Affiliations:** 1 School of Chemistry, Dalian University of Technology, Dalian 116024, China; 2 Institute of High Energy Physics, Chinese Academy of Sciences, Beijing 100049, China; 3 School of Nuclear Science and Technology, University of Chinese Academy of Sciences, Beijing 100049, China

We are delighted to dedicate this Special Issue to a leading figure in actinoid chemistry, Prof. Zhifang Chai, who devotes his whole career to the fundamental research and applications of radio- and nuclear chemistry. As a renowned radioanalytical chemist, he received the George von Hevesy Award in 2005 and was elected to be an academician of the CAS. His scientific achievements have been surveyed in a recent editorial of the journal *Chinese Chemical Letters* [1]. Here, we have the honor to share his contribution to the formulation of the development strategy of radiochemistry in China.

(1) Chairing the compilation of the Strategy of Chinese Discipline Development: Radiochemistry

At the request of the Chinese Academy of Sciences, Prof. Chai organized a committee to initiate the strategic study of the future development of radiochemistry. Under the leadership and coordination of Prof. Chai, tens of senior scholars and experts in various branches of radiochemistry joined the project. In this comprehensive book [2], an in-depth analysis of the current status of the development of radiochemistry was conducted, and the challenges faced by various branches of radiochemistry, including fission energy radiochemistry, fusion energy radiochemistry, environmental radiochemistry, radiopharmaceutical chemistry, radioanalytical chemistry, nuclear chemistry, radiochemistry and interdisciplinary fields, radiochemistry in national security, radiochemistry databases, and radiochemistry education, and potential solutions were studied. This book (Figure 1, left) provides a valuable consultation for the Chinese government to formulate a policy for the long-term development of radiochemistry to promote scientific and technological innovation and applications.

(2) Chairing the compilation of the Encyclopedia of China: Nuclear Techniques.

At the request of the Editorial Committee of the Chinese Encyclopedia, Prof. Chai assumed the responsibility as the chief editor and organized a committee to edit the volume of nuclear techniques [3] from scratch, which was not included in the previous two editions of the Encyclopedia of China. Under the leadership of Prof. Chai, hundreds of professionals from universities and institutions actively participated in the project and contributed to the writing of the volume (Figure 1, right).

In the form of encyclopedia entries, this volume provides a comprehensive introduction of the discipline of nuclear techniques, including the knowledge system, fundamental concepts and theories, important figures, representative works, and institutions. It covers the fundamental knowledge and research achievements in the field of nuclear science and techniques and offers a precious reference book for researchers owing to its scientific rigorousness and its completeness in content.

(3) Fostering the renaissance of the discipline of radiochemistry in China

The dawn of the new century has witnessed a hard time for the discipline of nuclear science owing to the shortage of financial support and job opportunities that shadow the development of research and the enrollment of promising students [4]. After entering the new century, worldwide concerns regarding energy resources and the environment motivated an international campaign to turn to sustainable energy solutions, which included the civil application of nuclear energy. Prof. Chai seized this opportunity and actively coordinated domestic peer scientists to adapt the discipline of the development of radiochemistry to the new situation by proposing a novel understanding of how the discipline can serve the sustainable development of science and society.

In order to promote the revitalization of radiochemistry, Prof. Chai and his colleagues organized a series of symposiums to discuss and formulate the development plan for the radiochemistry discipline. Under the organization and coordination of Prof. Chai, the National Natural Science Foundation of China has successively hosted strategic seminars on radiochemistry since 2004 to chart the future development of the discipline of radiochemistry and promote the cultivation of radiochemistry talents in China. These endeavors have significantly boosted research and education in the field of radiochemistry. The number of institutions engaged in radiochemical research in China has surged from less than fifty to more than one hundred and fifty, and more and more young talents start their careers in fields related to nuclear science.

With this, we salute Prof. Zhifang Chai and dedicate this Special Issue to him to honor his contributions to the development of the discipline of radiochemistry.

## Figures and Tables

**Figure 1:**
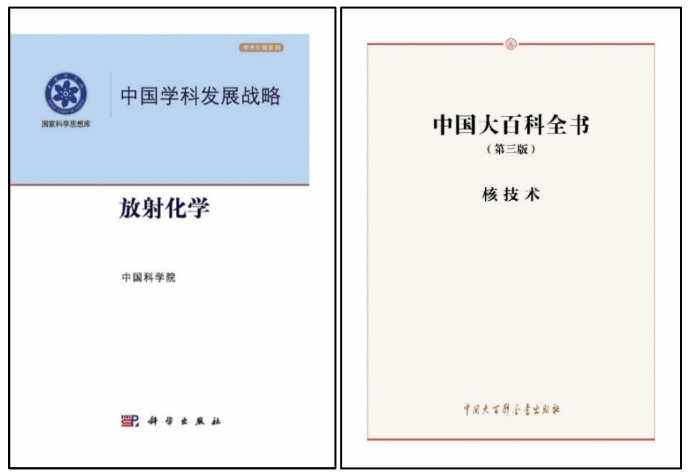
The cover pages of the two publications compiled by Prof. Chai and colleagues. (**left**) the Strategy of Chinese Discipline Development: Radiochemistry, (**right**) Encyclopedia of China: Nuclear Techniques.

## References

[1] Zhao Y., Chen C., Feng W., Zhang Z., Xu D., Shi W., Wang S., Li Y.-F. (2022). Professor Zhifang Chai: Scientific contributions and achievements. Chin. Chem. Lett..

[2] Chinese Academy of Sciences (2013). Development Strategy of Chinese Discipline: Radiochemistry.

[3] Chai Z.F. (2022). Encyclopedia of China (The 3rd Edition)—Nuclear Techniques.

[4] Wu W.-S. (2009). Nuclear and Radiochemistry Education and training in China. J. Nucl. Radiochem..

